# Benzothiadiazole, a plant defense inducer, negatively regulates sheath blight resistance in *Brachypodium distachyon*

**DOI:** 10.1038/s41598-018-35790-w

**Published:** 2018-11-26

**Authors:** Yusuke Kouzai, Yoshiteru Noutoshi, Komaki Inoue, Minami Shimizu, Yoshihiko Onda, Keiichi Mochida

**Affiliations:** 1 Bioproductivity Informatics Research Team, RIKEN Center for Sustainable Resource Science, 1-7-22 Suehiro-cho, Tsurumi, Yokohama, 230-0045 Japan; 2 Kihara Institute for Biological Research, Yokohama City University, 641-12 Maioka-cho, Totsuka, Yokohama, 244-0813 Japan; 3 Graduate School of Environmental and Life Science, Okayama University, 1-1-1 Tsushimanaka, Okayama, 700-8530 Japan; 4 Institute of Plant Science and Resources (IPSR), Okayama University, 2-20-1 Chuo, Kurashiki, 710-0046 Japan; 5 Microalgae Production Technology Laboratory, RIKEN Baton Zone Program, RIKEN Cluster for Science, Technology and Innovation Hub, 1-7-22 Suehiro-cho, Tsurumi-ku, Yokohama, Kanagawa 230-0045 Japan; 6 Graduate School of Nanobioscience, Yokohama City University, 22-2 Seto, Kanazawa-ku, Yokohama, Kanagawa 236-0027 Japan

## Abstract

Plant defense inducers that mimic functions of the plant immune hormone salicylic acid (SA) often affect plant growth. Although benzothiadiazole (BTH), a synthetic analog of SA, has been widely used to protect crops from diseases by inducing plant defense responses, we recently demonstrated that SA, but not BTH, confers resistance against *Rhizoctonia solani*, the causal agent of sheath blight disease, in *Brachypodium distachyon*. Here, we demonstrated that BTH compromised the resistance of Bd3-1 and Gaz4, the two sheath blight-resistant accessions of *B*. *distachyon*, which activate SA-dependent signaling following challenge by *R*. *solani*. Moreover, upon analyzing our published RNA-seq data from *B*. *distachyon* treated with SA or BTH, we found that BTH specifically induces expression of genes related to chloroplast function and jasmonic acid (JA) signaling, suggesting that BTH attenuates *R*. *solani* resistance by perturbing growth-defense trade-offs and/or by inducing a JA response that may increase susceptibility to *R*. *solani*. Our findings demonstrated that BTH does not work as a simple mimic of SA in *B*. *distachyon*, and consequently may presumably cause unfavorable side effects through the transcriptional alteration, particularly with respect to *R*. *solani* resistance.

## Introduction

Salicylic acid (SA) is a phytohormone that plays a central role in plant disease resistance against fungi, oomycetes, bacteria, and viruses^1^. It is rapidly biosynthesized in response to pathogen challenges and invokes various defense responses, including hypersensitive response, defense gene expression, phytoalexin biosynthesis, and systemic acquired resistance^1^. Mutations in genes involved in SA biosynthesis or in the signaling pathway downstream of SA enhance the susceptibility of plants to diseases^2,3^. Exogenous application of SA also induces defense responses and confers disease resistance to treated plants. The effects of SA on plant disease resistance often depend on the plant species and the pathogen’s lifestyle. For examples, in tomato, SA increases susceptibility to the necrotrophic pathogen *Botrytis cinerea* by antagonizing the signaling pathway of jasmonic acid (JA), another defense-related phytohormone^4^, whereas in *Arabidopsis*, both SA and JA are required for *B*. *cinerea* resistance^5^. To capitalize on the properties of SA, chemical inducers of the SA-signaling pathway have been developed and used as plant defense inducers in practical crop protection^6,7^. Benzothiadiazole (BTH), also referred to as acibenzolar-*S*-methyl, is a synthetic chemical analog of SA that has been used for protection against diseases in various agronomically important crops, such as rice, wheat, potato, and tomato^8–11^. BTH is believed to mimic the effects of SA, because transcriptional changes induced by SA closely mirror those induced by BTH, which are both under the control of Nonexpressor of Pathogenesis-Related 1 (NPR1), a master regulator of the SA-signaling pathway, in *Arabidopsis*^12,13^.

Care needs to be taken with respect to the use of plant defense inducers because SA has been shown to affect plant growth^14^. In particular, marked activation of the defense responses induced by SA is often associated with negative effects on plant growth. For example, *cpr* and *slh1* mutants of *Arabidopsis*, which accumulate high levels of SA, show severe growth defects^15,16^. Moreover, high doses of SA and related plant defense inducers negatively affect photosynthesis, growth, and seed production in plants^17,18^. Conversely, *Arabidopsis* mutants and transgenic lines that are deficient in endogenous SA show increased growth and seed production^19^. These examples demonstrate that growth-defense trade-offs often influence plant defense responses resulting from the allocation of limited resources. Because plant defense requires massive amounts of energy to regulate the various cellular processes involved in immune responses, plants presumably deploy a trade-off system between defense and growth to effectively combat pathogens using limited resources^20,21^.

Sheath blight, caused by the soil-borne plant pathogenic fungus *Rhizoctonia solani* AG-1, IA, is one of the most destructive diseases of cultivated rice^22^. It occurs in areas of rice production areas worldwide and is particularly prevalent in the paddy fields of East Asia as well as the southern United States^22^. We previously reported that SA-dependent plant immunity contributes to sheath blight resistance in rice and the small model grass species *Brachypodium distachyon*^23^. Exogenous SA treatment induced resistance to *R*. *solani* resistance in a sheath blight-susceptible *B*. *distachyon* accession, Bd21. In contrast, treatment with JA enhanced the susceptibility of Bd21 to *R*. *solani*. Surprisingly, treatment with BTH could not increase the level of *R*. *solani* resistance in Bd21^23^. Our comparative transcriptome analysis of Bd21 leaves after treatment with SA or BTH revealed that their transcriptional changes involved a number of common genes but also included very large differences although the corresponding effects on gene expression in *Arabidopsis* following treatment with either SA or BTH were very similar^13^. Specifically, SA induced the expression of 89 genes that were not induced by BTH in *B*. *distachyon*, including a number of genes related to secondary cell wall (SCW) biosynthesis. SCW reinforcement, such as lignification and suberization, acts as a physical barrier that prevents the access of pathogens to host cells^24^. Therefore, we speculated that the lack of transcriptional activation of SCW-related genes may explain why BTH cannot confer *R*. *solani* resistance in *B*. *distachyon*^23^. On the other hand, the number of differentially expressed genes (DEGs) in *B*. *distachyon* following treatment with BTH was 2.8 times greater than that with SA, and BTH specifically regulated the expression of more than 1,300 genes at the same BTH concentration as did SA^23^. Of the DEGs for SA, 82% were also regulated by BTH but this figure corresponds to only 29% of the total number of DEGs detected following treatment with BTH^23^. These findings indicate that, in *B*. *distachyon*, BTH affects various other cellular responses in addition to the SA-dependent signaling in *B*. *distachyon*. It is also possible that the BTH-specific DEGs include genes that negatively regulate resistance of *B*. *distachyon* to *R*. *solani*.

In this study, we further investigated the reasons why SA, but not BTH, induces resistance to *R*. *solani* in *B*. *distachyon*. We found that BTH treatment induced susceptibility to *R*. *solani* in two sheath blight-resistant accessions of *B*. *distachyon*, indicating that BTH*-*induced cellular responses included negative impacts on *R*. *solani* resistance. Analysis of our previously published RNA-seq data of *B*. *distachyon* treated with SA or BTH identified potential host cellular responses that were induced by BTH that could attenuate the defense responses to *R*. *solani*. Our results demonstrated that, whereas BTH works as a simple mimic of SA in *Arabidopsis*, this is not the situation in *B*. *distachyon*, with BTH treatment of *B*. *distachyon* being associated with unfavorable side effects, particularly with respect to *R*. *solani* resistance.

## Results

### BTH does not induce *R*. *solani* resistance in the sheath blight-susceptible *B*. *distachyon* accession Bd21 at low concentrations

In our previous study^23^, we demonstrated that treatment of *B*. *distachyon* accession Bd21 with 500 μM BTH treatment did not induce resistance to *R*. *solani*. To investigate whether this lack of effect was due to the high BTH concentration used, we assessed the effect of a range of low concentrations of BTH on resistance to *R*. *solani*. We treated detached Bd21 leaves with 1, 10, 100, or 500 μM BTH and 24 h later inoculated them with *R*. *solani*. The results showed that BTH did not induce resistance at any of the concentrations tested (Supplementary Fig. S1a,b), although the expression of a SA-marker gene *BdWRKY45L1*^25^ was induced even at the lowest concentration of 1 μM (Supplementary Fig. S1c). These results indicate that the absence of an ability of BTH to induce *R*. *solani* resistance is not due to a side effect observed only at a high BTH concentration.

### BTH compromises *R*. *solani* resistance in two sheath blight-resistant accessions of *B*. *distachyon*

**Figure 1:**
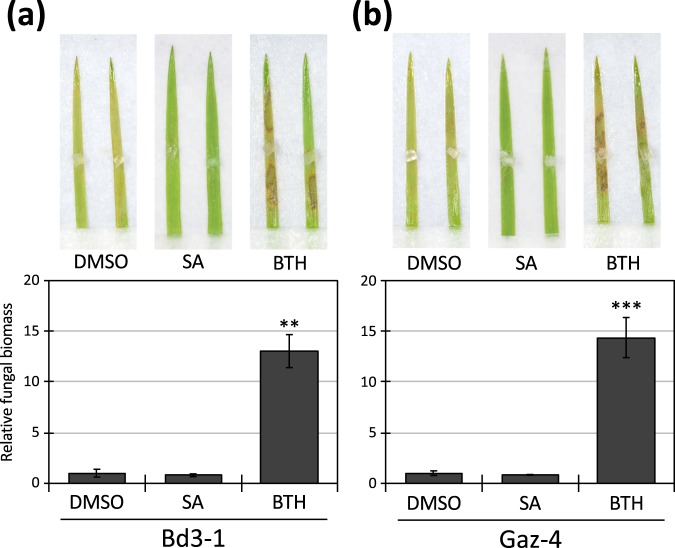
BTH compromises *Rhizoctonia solani* resistance in *Brachypodium distachyon* accessions Bd3-1 and Gaz-4. Detached leaves of *B*. *distachyon* Bd3-1 and Gaz-4 were spray-treated with 0.5% (v/v) DMSO, 0.5 mM SA, or 0.5 mM BTH and 24 h later inoculated with *R*. *solani* mycelial plugs (2–3 mm^3^). Lesion formation (upper panel) and relative biomass of *R*. *solani* (lower panel) in (**a**) Bd3-1 and (**b**) Gaz-4 leaves were evaluated at 3 d post-inoculation. Data are presented as means ± SEM of values relative to the DMSO treatment, *n* = 9; **P < 0.01, ***P < 0.001 using Student’s *t*-tests. The experiments were performed twice with similar results and a representative result is shown.

To investigate the cause of the difference between SA and BTH in terms of inducing resistance to *R*. *solani*, we used *B*. *distachyon* accessions Bd3-1 and Gaz-4. Bd21 is susceptible to *R*. *solani*, whereas Bd3-1 and Gaz-4 are resistant to this pathogen and activate SA-dependent signaling following *R*. *solani* inoculation^23^. The detached leaves of Bd3-1 and Gaz-4 were treated with SA or BTH and subsequently inoculated with *R*. *solani*. We found that SA treatment did not significantly affect the *R*. *solani* resistance in either accession at 3 d post-inoculation (dpi) (Fig. 1). This suggests that *R*. *solani* resistance in these accessions could be conferred by a gene set similar to those induced by SA treatment or that SA-induced responses may be able to work additively with the *R*. *solani* resistance in these accessions. Intriguingly, BTH treatment compromised *R*. *solani* resistance in both accessions, as evidenced by increases in both lesion formation and foliar fungal biomass (Fig. 1). To test whether BTH directly promoted fungal growth, we measured the diameter of colonies of *R*. *solani* on PDA medium containing either SA or BTH. As we reported earlier^23^, SA did not affect the growth of *R*. *solani* on PDA medium, but BTH inhibited its growth under our experimental conditions (Supplementary Fig. S2). BTH has been shown to have no antimicrobial activity against various plant pathogenic fungi *in vitro*^8^, so these observed effects on colony growth may be due to the experimental conditions. Indeed, BTH itself did not promote hyphal growth of *R*. *solani* on PDA medium, indicating that some of the cellular responses in *B*. *distachyon* triggered by BTH act negatively on the *R*. *solani* resistance even in accessions that express the SA-dependent defense phenotype against *R*. *solani*, such as Bd3-1 and Gaz-4.

### BTH induces biological functions related to chloroplast properties and jasmonic acid response in *B*. *distachyon*

**Figure 2:**
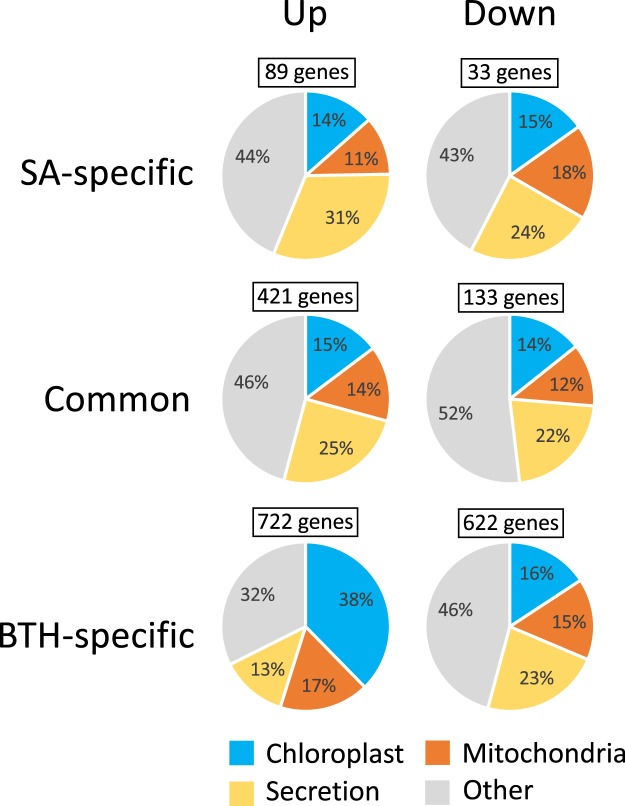
Predicted subcellular localization of proteins corresponding to the sets of differentially expressed genes after treatment with SA or BTH in *Brachypodium distachyon*. Subcellular localization of the proteins corresponding to each gene set was predicted using TargetP analysis. Proteins were predicted to localize to chloroplasts, mitochondria, secretion pathways, or other locations.

**Figure 3:**
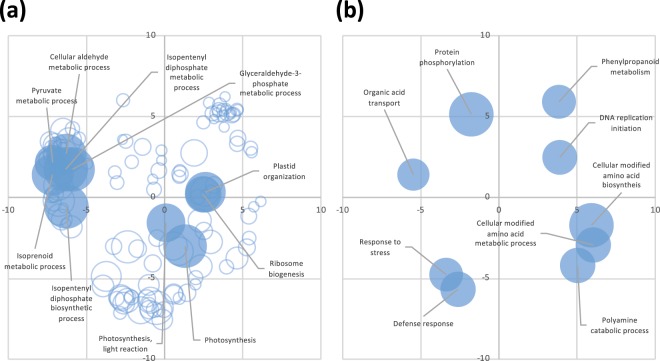
The GO enrichment analysis for the sets of genes specifically regulated by BTH in *Brachypodium distachyon*. GO enrichment analysis for the BTH-specific upregulated and downregulated genes were performed using AgriGO^47^. The significantly over-represented GO terms categorized to biological process in each gene set were summarized and visualized in two-dimensional semantic similarity-based scatter plots using REVIGO^27^. Panel a shows the summarized GO terms for the “BTH-specific upregulated genes.” Panel b shows the summarized GO terms for the “BTH-specific downregulated genes.” The circle size represents the −log_10_ transformed FDR value. Circles depicted by filled color with references show the top 10 significantly over-represented GO terms for each gene set.

To understand which host responses account for the suppressive effect of BTH on *R*. *solani* resistance, we analyzed the published RNA-seq data from *B*. *distachyon* leaves of the sheath blight-susceptible accession Bd21 treated with SA or BTH for 24 h (DDBJ accession no. DRP003562)^23^. In our previous study, we had classified the DEGs in response to SA or BTH into six gene sets: SA-specific genes, commonly regulated genes (i.e., SA and BTH), and BTH-specific genes, with each subdivided into either up- or down-regulated genes (Fig. 2). In the current study, we further subdivided each set by the subcellular localizations of the proteins that they encode, which were predicted using the TargetP program^26^. A comparison of the proportions determined by these analyses clearly demonstrated that the BTH-specific upregulated genes contained a larger number of genes encoding chloroplast-targeted proteins (38% of the BTH-specific genes) than did the other gene sets (14–16%) (Fig. 2). No characteristic patterns with respect to subcellular localization were found in the BTH-specific downregulated genes (Fig. 2). In addition, we performed a Gene Ontology (GO) enrichment analysis for the BTH-specific upregulated and downregulated genes and identified 323 and 36 significantly over-represented GO terms, respectively, when applying a threshold false discovery rate (FDR) value lower than 0.05 (Supplementary Tables S1 and S2). To represent the functional properties of the BTH-specific genes, the markedly over-represented GO terms categorized to biological process were summarized and depicted in scatter plots based on their semantic similarities (Fig. 3), using REVIGO^27^. Consistent with the high proportion of chloroplast-localized proteins shown in Fig. 2, GO terms related to chloroplast properties, such as photosynthesis (GO:0015979, FDR value: 5.3E^−25^; GO:0019684, FDR value: 8.5E^−20^) and the non-mevalonate pathway (GO:0046490, FDR value: 5.3E^−26^; GO:0009240, FDR value: 5.3E^−26^; GO:0019682, FDR value: 5.3E^−26^; GO:0006090, FDR value: 8.8E^−17^) were over-represented in the BTH-specific upregulated genes (Fig. 3a). These results imply that BTH treatment could upregulate chloroplast activities, including photosynthesis, in *B*. *distachyon*. In the BTH-specific downregulated genes, GO terms related to cellular amino acid modification (GO:0042398, FDR value: 1.3E^−03^; GO:0006468, FDR value: 1.3E^−03^; GO:0006575, FDR value: 1.5E^−02^), and stress responses (GO:0006952, FDR value: 1.5E^−02^; GO:0006950, FDR value: 2.2E^−02^) were over-represented (Fig. 3b).

We also focused on the phytohormone-related GO terms and found that biological functions related to SA, JA, ethylene (ET), abscisic acid (ABA), and auxin (AUX) were all over-represented in the BTH-specific upregulated genes (Table 1). This indicates that BTH can affect biological processes related to not only SA but also other phytohormones in *B*. *distachyon*. Five GO terms related to JA were over-represented in this gene set: biosynthetic process (GO:0009695, 15 genes, FDR value: 5.70E^−04^), metabolic process (GO:0009694, 15 genes, FDR value: 2.50E^−03^), and the downstream signaling pathway (GO:0009753, 29 genes, FDR value: 7.50E^−04^; GO:0009867, 23 genes, FDR value: 3.00E^−04^; GO:0071395, FDR value: 3.00E^−04^). Moreover, two GO terms related to oxylipins were also over-represented in this gene set: biosynthetic process (GO:0031408, 15 genes, FDR value: 9.80E^−04^) and metabolic process (GO:0031407, 16 genes, FDR value: 1.60E^−03^) (Supplementary Table S1). The numbers of GO terms and annotated genes related to SA (6 GO terms, 134 genes) and JA (5 GO terms, 105 genes) were clearly greater than those related to ET (1 GO term, 19 genes), ABA (2 GO terms, 33 genes), and AUX (1 GO term, 20 genes) in this gene set (Table 1). Although signal crosstalk between SA and ET, ABA, or AUX are known in rice and *Arabidopsis*^28,29^, GO terms related to the phytohormones ET, ABA and AUX were not detected when a threshold FDR value lower than 0.01 was applied (Table 1). These results indicated that BTH activates certain signaling pathways of both SA and JA in *B*. *distachyon*. In contrast, no GO terms related to phytohormones were identified in the BTH-specific downregulated genes.

**Table 1:** Phytohormone-related functions (biological process) over-represented in the BTH-specific upregulated genes of *Brachypodium distachyon*.

GO accession	GO name	FDR	Number of annoteted genes in the query set	Number of annoteted genes in the background set	Percentage of annoteted genes in the query set	Percentage of annotated genes in the background set	Related phytohormones
GO:0009696	salicylic acid metabolic process	8.10E-05	20	113	3.34	1.00	SA
GO:0009862	systemic acquired resistance, salicylic acid mediated signaling pathway	1.70E-04	21	129	3.51	1.14	SA
GO:0009867	jasmonic acid mediated signaling pathway	3.00E-04	23	155	3.84	1.37	JA
GO:0071395	cellular response to jasmonic acid stimulus	3.00E-04	23	155	3.84	1.37	JA
GO:0009697	salicylic acid biosynthetic process	3.10E-04	18	104	3.01	0.92	SA
GO:0009751	response to salicylic acid stimulus	3.50E-04	29	224	4.84	1.97	SA
GO:0009695	jasmonic acid biosynthetic process	5.70E-04	15	80	2.50	0.70	JA
GO:0009753	response to jasmonic acid stimulus	7.50E-04	29	234	4.84	2.06	JA
GO:0009863	salicylic acid mediated signaling pathway	1.90E-03	23	176	3.84	1.55	SA
GO:0071446	cellular response to salicylic acid stimulus	1.90E-03	23	176	3.84	1.55	SA
GO:0009694	jasmonic acid metabolic process	2.50E-03	15	92	2.50	0.81	JA
GO:0009733	response to auxin stimulus	2.60E-02	20	178	3.34	1.57	AUX
GO:0071215	cellular response to abscisic acid stimulus	3.00E-02	17	143	2.84	1.26	ABA
GO:0009723	response to ethylene stimulus	3.40E-02	19	170	3.17	1.50	ET
GO:0009738	abscisic acid mediated signaling pathway	3.90E-02	16	135	2.67	1.19	ABA

### BTH induces the expression of chloroplast- and JA-related genes in *B*. *distachyon*

**Figure 4:**
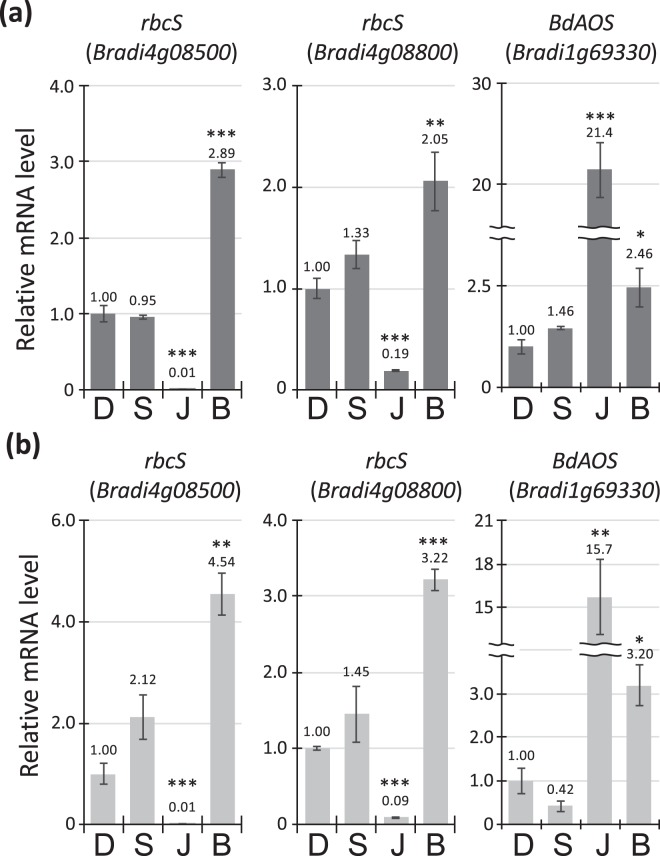
BTH induces expression of chloroplast- and JA-related genes in *Brachypodium distachyon* Bd21 and Bd3-1. Detached leaves of *B*. *distachyon* were spray-treated with 0.5% (v/v) DMSO, 0.5 mM SA, 0.5 mM JA, or 0.5 mM BTH for 24 h. Expression levels of *rbcS* genes and *BdAOS* were evaluated by qRT-PCR analysis in Bd21 (**a**) and Bd3-1 (**b**). Data are presented as means ± SEM of values relative to the DMSO treatment, *n* = 4; *P < 0.05, **P < 0.01, ***P < 0.001 using Student’s *t*-tests. The experiments were performed twice with similar results and a representative result is shown.

To confirm our observations from the transcriptome analysis, that BTH can induce the expression of chloroplast- and JA-related genes in *B*. *distachyon*, we investigated the expression of genes encoding allene oxide synthase (AOS) and the small subunit of ribulose-1,5-bisphosphate carboxylase/oxygenase (rbcS) in response to BTH, SA, or JA. The rbcS proteins are localized to the chloroplast and play key roles in plant photosynthesis^30^. In *B*. *distachyon*, two rbcS proteins encoded by *Bradi4g08500* and *Bradi4g08800*, have been identified in abundance in the soluble proteins extracted from young leaves^31^. AOS is required for the plant JA biosynthetic pathway^32^, and expression of *BdAOS* (*Bradi1g69330*), the closest homolog of rice *AOS2* in *B*. *distachyon*, is strongly responsive to JA^25^. In accordance with the results from our GO analysis of the BTH-specific upregulated genes, we found that transcription of these genes in accession Bd21 was significantly upregulated by BTH, but not by SA, in Bd21 (Fig. 4a). Similar to a previous observation in barley^33,34^, we also found that JA treatment decreased expression of the rbcS genes (Fig. 4a). Moreover, our gene expression analysis demonstrated that the induction of the genes was retained in the sheath blight-resistant accession Bd3-1 (Fig. 4b). These results support the hypothesis that BTH treatment can activate genes related to both chloroplast and JA in *B*. *distachyon*.

## Discussion

Our previous study had demonstrated that treatment with SA, but not BTH, induced *R*. *solani* resistance in a sheath blight-susceptible *B*. *distachyon* accession, Bd21, although BTH is widely used as a SA analog and it regulated more than 80% of the DEGs identified following SA treatment in Bd21^23^. Here we showed that BTH treatment also suppressed the *R*. *solani* resistance in two sheath blight-resistant accessions of *B*. *distachyon*, Bd3-1 and Gaz-4, possibly by affecting the host-dependent cellular responses (Fig. 1 and Supplementary Fig. S2). From this result, we conclude that the DEGs specifically regulated in response to BTH treatment include genes that decrease the disease resistance of *B*. *distachyon* to *R*. *solani*. Because BTH did not enhance susceptibility to *R*. *solani* at any tested concentration in Bd21 that did not activate SA-dependent signaling against *R*. *solani* inoculation (Supplementary Fig. S1), the BTH-specific DEGs may particularly disturb the SA-dependent resistance against *R*. *solani*, which is exhibited by the *B*. *distachyon* accessions Bd3-1 and Gaz-4. Indeed, SA-dependent signaling is rapidly activated following inoculation of Bd3-1 and Gaz-4, but not Bd21with *R*. *solani*^23^.

Our analysis of the RNA-seq data of Bd21 leaves treated with SA or BTH provided two possible reasons why BTH did not confer *R*. *solani* resistance in sheath blight-susceptible *B*. *distachyon*. One is related to chloroplast activities. The BTH-specific upregulated genes include a large number of genes encoding chloroplast-targeted proteins (Fig. 2) and are significantly over-represented with respect to functions related to chloroplast activities such as photosynthesis (Fig. 3). These findings are supported by our qRT-PCR-based expression analysis of the *rbcS* genes in response to BTH treatment of Bd21 and Bd3-1 (Fig. 4). In contrast to our findings, BTH had previously been reported to downregulate genes involved in photosynthesis via an NPR1-dependent pathway in *Arabidopsis* and rice^13,35^. Rice plants reduced the maximum quantum yield of photosystem II (Fv/Fm) after treatment with BTH^35^. This down-regulation of photosynthesis could be caused by a trade-off between defense and growth. The transfer of resources from growth to defense is known to be a part of plant defense responses^21,36,37^. An example of this has been reported with respect to photosynthetic electron transport, which was reduced within a few hours in response to infection of tobacco with an incompatible strain of *Phytophthora nicotianae*^38^. Moreover, reduction in photosynthetic activities was found to be more rapid and pronounced after inoculation of *Arabidopsis* with an incompatible strain of *Pseudomonas syringae* pv. *tomato* DC3000 than that with a compatible strain^39^. Considering the switch in resource allocation from growth to defense during disease resistance responses^21^, it is possible that the BTH-induced up-regulation of chloroplast activities negatively affects the expression of disease resistance in *B*. *distachyon*, particularly against *R*. *solani* infection. The question remains as to whether the BTH-induced stimulation of chloroplast activity is specific to only *B*. *distachyon* because BTH upregulated the expression of chloroplast-related genes in *B*. *distachyon* but not in rice^35^. The plant immune system is known to differ between monocots and dicots^25,28,40^. In *Arabidopsis*, the transcriptional changes in response to BTH treatment are similar to those which occur in response to SA^12,13^. However, we demonstrated that BTH affects the expression of more genes in *B*. *distachyon* than SA does^23^. As far as we know, the only comparative transcriptome analysis of monocot in response to both SA and BTH was reported in *B*. *distachyon*^23^, although transcriptome profiling in response to BTH has been reported in rice^11,35^. Rice plants naturally contain a much higher level of endogenous SA than do other monocots, including *B*. *distachyon* and barley^23,41–43^. Therefore, it is possible that rice has acquired unique features, particularly with respect to biotic defense response, during domestication and breeding, compared with other monocots. Further analysis is needed to clarify whether the BTH-induced up-regulation of chloroplast-related genes is specific to *B*. *distachyon* or is conserved in other monocots.

The other possible reason why BTH does not induce *R*. *solani* resistance in *B*. *distachyon* is JA-related responses. The functional enrichment analysis of the BTH-specific upregulated genes demonstrated that BTH can affect a broad range of phytohormone signaling pathways in *B*. *distachyon*, particularly those associated with SA and JA (Table 1). In fact, *BdAOS*, a marker gene for JA response^25^, was significantly upregulated by BTH treatment in both susceptible and resistant accessions Bd21 and Bd3-1 (Fig. 4). In accordance with our findings, BTH has been reported to activate JA signaling in rice^40^. A comparative transcriptome analysis of BTH- and JA-treated rice demonstrated that more than half of the BTH-induced genes are also upregulated by JA^40^, suggesting that the induction of JA-related biological functions by BTH should be a common feature in rice and *B*. *distachyon*. In rice, BTH treatment enhanced the resistance against both *Magnaporthe oryzae* and *Xanthomonas oryzae* pv. *oryzae* (*Xoo*)^11,44^, which are the causal agents of blast disease and bacterial leaf blight, respectively. This is consistent with the result that exogenously applied JA also improves rice resistance against these pathogens^45,46^. SA and JA are known to induce the expression of the same gene set, designated as a common defense pathway^25,28,40^, and this gene set should contribute to defense against these pathogens. In contrast, in *B*. *distachyon*, treatment with either JA or BTH increases susceptibility to *R*. *solani* in both a sheath blight-susceptible accession^23^ as well as two sheath blight-resistant accessions (Fig. 1). Taking these findings together, the JA-related biological functions induced by BTH should act positively on the resistance against *M*. *oryzae* and *Xoo*, but negatively on the resistance against *R*. *solani*. These differences may be attributable to the infection strategy of each pathogen.

There are some common transcriptional changes in the responses of the model monocot plant *B*. *distachyon* to SA and BTH, but many more different ones, a finding which contrasts with the situation in *Arabidopsis*. Although the transcriptome profile in response to SA remains unclear in another model monocot, rice, our results showed that the actions of plant defense inducers as well as phytohormones are not always the same among different plant species. We need to be careful about such differences because they could be associated with unexpected effects on non-target pathogens. Characterization of plant defense inducers by comparative transcriptome analyses will facilitate the evidence-based design of crop protection chemicals for durable and sustainable crop protection, as well as providing new insights into the evolution of the SA signaling pathway in plants.

## Methods

### Plant and fungal materials and growth conditions

Dry seeds of the *B*. *distachyon* accessions Bd21, Bd3-1, and Gaz-4 were provided by the National Plant Germplasm System of USDA-ARS. The seeds were sown on moist filter paper, incubated at 4 °C in the dark for 3 d, and then germinated in a growth chamber (MLR-350HT; Sanyo, Osaka, Japan) at 25 °C under a photoperiod of 16 h light and 8 h dark for 3 d. The germinated seedlings were transplanted to 24-well microplates filled with soil (Sakata Supermix-A; Sakata Seed, Yokohama, Kanagawa, Japan). The seedlings were subsequently grown in a growth chamber (Nippon Medical & Chemical Instruments, Osaka, Japan) at 23 °C under a photoperiod of 20 h light and 4 h dark for 3 wk until the experiments carried out.

The *R*. *solani* AG-1, IA strain MAFF305230 was obtained from the Genebank of the National Agricultural Research Organization (NARO) in Japan and grown on potato dextrose agar (PDA; BD, Franklin Lakes, NJ, USA) plates at 23 °C for 3–5 d. To examine the effect of SA and BTH on fungal growth, mycelial plugs (3 mm diameter) bored from the edge of the fungal mycelia growing on PDA plates were inoculated onto the center of PDA plates containing 0.5% (v/v) dimethyl sulfoxide (DMSO), 0.1 mM SA in 0.5% (v/v) DMSO, or 0.1 mM BTH in 0.5% (v/v) DMSO and incubated at 23 °C for 24 h.

### Chemicals

Sodium salicylate (SA) (Wako, Osaka, Japan) and methyl jasmonate (JA) (Wako) were used as phytohormones. Benzothiadiazole (acibenzolar-*S*-methyl, BTH) (Wako) was used as a functional analog of SA. These were diluted with DMSO (Wako).

### Analysis of RNA-seq data

The previously published RNA-seq data from *B*. *distachyon* Bd21 leaves obtained 24 h after treatment with SA or BTH (DDBJ accession no. DRP003562)^23^ were used. The subcellular localization of the proteins corresponding to a set of DEGs was analyzed using TargetP software^26^. The proteins were predicted to be located in mitochondria, chloroplasts, secretory pathways, or other sites based on their N-terminal amino acid sequence. GO enrichment analysis for the set of DEGs was performed as follows. GO terms were assigned to *B*. *distachyon* genes based on the GO annotations of homologs most similar to those of *Arabidopsis*, as identified in a blastp analysis. Significantly enriched GO terms categorized to biological processes with a threshold of FDR < 0.05 from Fisher’s exact test were identified by AgriGO analysis^47^. Summarization of GO terms according to their semantic similarities and the representation of summarized GO terms in 2D semantic space were carried out using the REVIGO web service^27^.

### Inoculation tests

Detached leaves of *B*. *distachyon* were placed on moist filter paper in a square plate. After 1 d wound acclimation, the leaves were spray-treated with chemical solution (0.5% [v/v] DMSO, SA in 0.5% [v/v] DMSO, or BTH in 0.5% [v/v] DMSO) containing 0.04% (v/v) Tween 20 and incubated at 23 °C under a photoperiod of 20 h light and 4 h dark for 24 h. Before subsequent experiments were carried out, droplets of the chemical solution attached to the leaf surface were removed. The leaves were then inoculated with cubic mycelial plugs (2–3 mm^3^) of *R*. *solani* and incubated at 23 °C under a photoperiod of 20 h light and 4 h dark for 3 d. Disease severity was evaluated from the amounts of fungal DNAs in the inoculated leaves, expressed as fungal biomass. Total DNAs were extracted from the inoculated leaves using a Nucleospin Plant II Kit (Takara Bio, Shiga, Japan), and qPCR for fungal DNAs (28 S rDNA) was performed using a SYBR Premix Ex Taq II (Takara Bio) employing the Applied Biosystems 7500 System (Thermo Fisher Scientific, Waltham, MA, USA). The *B*. *distachyon BdFIM* gene (*Bradi2g13800*) was used to normalize the data. The primers used for amplification are listed in Supplementary Table S3.

### Gene expression analysis

Total RNAs were extracted from *B*. *distachyon* leaves following 24 h treatment with chemical solution (0.5% [v/v] DMSO, SA in 0.5% [v/v] DMSO, JA in 0.5% [v/v] DMSO, or BTH in 0.5% [v/v] DMSO) using a RNeasy Plant Mini Kit (Qiagen, Hilden, Germany) and cDNAs were synthesized using an Omniscript RT Kit (Qiagen). Gene expression analysis for *BdWRKY45L1*, *BdAOS*, and *rbcS* genes was performed by qRT-PCR using SYBR Premix Ex Taq II (Takara Bio) in conjunction with the Applied Biosystems 7500 System (Thermo Fisher Scientific). The *BdUbi4* gene (*Bradi3g04730*) was used to normalize the data. The primers used for amplification are listed in Supplementary Table S3.

### Statistical analysis

Statistical differences between populations were analyzed using Student’s *t*-tests. Data are presented as the mean value and error bars show the standard error (SE).

## Electronic supplementary material

Supplementary Information

## Data Availability

DNA Data bank of Japan (DDBJ) DRP003562 (2017). https://trace.ddbj.nig.ac.jp/DRASearch/study?acc=DRP003562.
