# Supplementary material for: Benzothiadiazole, a plant defense inducer, negatively regulates sheath blight resistance in Brachypodium distachyon
Source: Sci Rep. 2018 Nov 26;8:17358. doi: 10.1038/s41598-018-35790-w (PMC6255916; doi:10.1038/s41598-018-35790-w)
Supplement: Supplementary file 1 — Supplementary Information [file 41598_2018_35790_MOESM1_ESM.pdf]

Benzothiadiazole, a plant defense inducer, negatively regulates sheath blight resistance in *Brachypodium distachyon*

Yusuke Kouzai[1,2], Yoshiteru Noutoshi[3], Komaki Inoue[1], Minami Shimizu[1,2], Yoshihiko Onda[1,2], Keiichi Mochida[1,2,4,5,6*]

1. Bioproductivity Informatics Research Team, RIKEN Center for Sustainable Resource Science, 1-7-22 Suehiro-cho, Tsurumi, Yokohama, 230-0045, Japan
2. Kihara Institute for Biological Research, Yokohama City University, 641-12 Maioka-cho, Totsuka, Yokohama, 244-0813, Japan
3. Graduate School of Environmental and Life Science, Okayama University, 1-1-1 Tsushimanaka, Okayama, 700-8530, Japan
4. Institute of Plant Science and Resources (IPSR), Okayama University, 2-20-1 Chuo, Kurashiki, 710-0046, Japan
5. Microalgae Production Technology Laboratory, RIKEN Baton Zone Program, RIKEN Cluster for Science, Technology and Innovation Hub, 1-7-22 Suehiro-cho, Tsurumi-ku, Yokohama, Kanagawa 230-0045, Japan.
6. Graduate School of Nanobioscience, Yokohama City University, 22-2 Seto, Kanazawa-ku, Yokohama, Kanagawa 236-0027, Japan.

*Corresponding author:
Keiichi Mochida
Tel: +81-(0)45-503-9183
Email: keiichi.mochida@riken.jp

Supplementary Information

Supplementary Table S1. GO terms over-represented in the BTH-specific upregulated genes
Supplementary Table S2. GO terms over-represented in the BTH-specific downregulated genes
Supplementary Table S3. Primers used in this study
Supplementary Figure S1. BTH does not induce *Rhizoctonia solani* resistance in *Brachypodium distachyon* Bd21 at low doses
Supplementary Figure S2. Effects of BTH on the mycelial growth of *Rhizoctonia solani*

Supplementary Table S1. GO terms over-represented in the BTH-specific upregulated genes

GO accession	GO name	Ontology	FDR	Number of annoteted genes in the query set	Number of annoteted genes in the background set	Percentage of annoteted genes in the query set	Percentage of annotated genes in the background set
GO:0044435	plastid part	Cellular component	8.70E-33	164	958	27.38	8.44
GO:0044434	chloroplast part	Cellular component	3.90E-31	158	939	26.38	8.27
GO:0009532	plastid stroma	Cellular component	2.40E-30	105	488	17.53	4.30
GO:0009570	chloroplast stroma	Cellular component	1.20E-26	97	472	16.19	4.16
GO:0009240	isopentenyl diphosphate biosynthetic process	Biological process	5.30E-26	62	204	10.35	1.80
GO:0019288	isopentenyl diphosphate biosynthetic process, mevalonate-indepenc	Biological process	5.30E-26	62	202	10.35	1.78
GO:0046490	isopentenyl diphosphate metabolic process	Biological process	5.30E-26	62	204	10.35	1.80
GO:0019682	glyceraldehyde-3-phosphate metabolic process	Biological process	5.30E-26	62	202	10.35	1.78
GO:0009579	thylakoid	Cellular component	3.40E-25	86	401	14.36	3.53
GO:0015979	photosynthesis	Biological process	5.30E-25	76	313	12.69	2.76
GO:0008299	isoprenoid biosynthetic process	Biological process	1.90E-23	75	323	12.52	2.85
GO:0006720	isoprenoid metabolic process	Biological process	1.50E-22	75	334	12.52	2.94
GO:0009941	chloroplast envelope	Cellular component	5.80E-22	84	429	14.02	3.78
GO:0009657	plastid organization	Biological process	8.90E-22	74	336	12.35	2.96
GO:0009526	plastid envelope	Cellular component	2.10E-21	85	447	14.19	3.94
GO:0006081	cellular aldehyde metabolic process	Biological process	4.40E-21	66	281	11.02	2.48
GO:0044436	thylakoid part	Cellular component	7.20E-21	67	302	11.19	2.66
GO:0009507	chloroplast	Cellular component	9.00E-21	266	2435	44.41	21.45
GO:0034357	photosynthetic membrane	Cellular component	1.30E-20	63	274	10.52	2.41
GO:0031976	plastid thylakoid	Cellular component	2.60E-20	70	336	11.69	2.96
GO:0009534	chloroplast thylakoid	Cellular component	2.60E-20	70	336	11.69	2.96
GO:0009536	plastid	Cellular component	3.00E-20	267	2478	44.57	21.83
GO:0031984	organelle subcompartment	Cellular component	3.20E-20	70	338	11.69	2.98
GO:0009535	chloroplast thylakoid membrane	Cellular component	7.40E-20	59	253	9.85	2.23
GO:0019684	photosynthesis, light reaction	Biological process	8.50E-20	60	249	10.02	2.19
GO:0055035	plastid thylakoid membrane	Cellular component	8.50E-20	59	254	9.85	2.24
GO:0042651	thylakoid membrane	Cellular component	8.80E-19	59	266	9.85	2.34
GO:0006364	rRNA processing	Biological process	3.20E-18	54	220	9.02	1.94
GO:0016072	rRNA metabolic process	Biological process	3.70E-18	54	221	9.02	1.95
GO:0042254	ribosome biogenesis	Biological process	1.00E-17	61	282	10.18	2.48
GO:0022613	ribonucleoprotein complex biogenesis	Biological process	3.40E-17	61	289	10.18	2.55
GO:0008654	phospholipid biosynthetic process	Biological process	8.80E-17	64	321	10.68	2.83
GO:0006090	pyruvate metabolic process	Biological process	8.80E-17	64	321	10.68	2.83
GO:0034660	ncRNA metabolic process	Biological process	3.50E-16	64	330	10.68	2.91
GO:0034470	ncRNA processing	Biological process	9.40E-16	56	267	9.35	2.35
GO:0009521	photosystem	Cellular component	1.30E-15	23	48	3.84	0.42
GO:0019637	organophosphate metabolic process	Biological process	5.30E-15	66	367	11.02	3.23
GO:0006644	phospholipid metabolic process	Biological process	7.90E-15	65	361	10.85	3.18
GO:0008610	lipid biosynthetic process	Biological process	2.60E-14	98	705	16.36	6.21
GO:0015995	chlorophyll biosynthetic process	Biological process	9.90E-14	32	105	5.34	0.93
GO:0031975	envelope	Cellular component	1.80E-13	89	654	14.86	5.76
GO:0031967	organelle envelope	Cellular component	1.80E-13	89	654	14.86	5.76
GO:0042180	cellular ketone metabolic process	Biological process	2.90E-13	148	1313	24.71	11.57
GO:0006779	porphyrin biosynthetic process	Biological process	3.80E-13	35	131	5.84	1.15
GO:0051188	cofactor biosynthetic process	Biological process	4.10E-13	56	307	9.35	2.70
GO:0005996	monosaccharide metabolic process	Biological process	6.00E-13	85	602	14.19	5.30
GO:0033014	tetrapyrrole biosynthetic process	Biological process	7.20E-13	35	134	5.84	1.18
GO:0043436	oxoacid metabolic process	Biological process	8.20E-13	145	1298	24.21	11.44
GO:0019752	carboxylic acid metabolic process	Biological process	8.20E-13	145	1298	24.21	11.44
GO:0006082	organic acid metabolic process	Biological process	8.40E-13	145	1299	24.21	11.44
GO:0009658	chloroplast organization	Biological process	2.40E-12	43	204	7.18	1.80
GO:0032787	monocarboxylic acid metabolic process	Biological process	4.60E-12	110	910	18.36	8.02
GO:0009295	nucleoid	Cellular component	5.20E-12	17	34	2.84	0.30
GO:0006091	generation of precursor metabolites and energy	Biological process	6.30E-12	71	480	11.85	4.23
GO:0051186	cofactor metabolic process	Biological process	8.00E-12	79	568	13.19	5.00
GO:0046148	pigment biosynthetic process	Biological process	1.40E-11	42	206	7.01	1.81
GO:0015994	chlorophyll metabolic process	Biological process	3.10E-11	36	160	6.01	1.41
GO:0006778	porphyrin metabolic process	Biological process	5.10E-11	39	188	6.51	1.66
GO:0033013	tetrapyrrole metabolic process	Biological process	6.00E-11	39	189	6.51	1.67
GO:0046394	carboxylic acid biosynthetic process	Biological process	1.40E-10	83	648	13.86	5.71
GO:0016053	organic acid biosynthetic process	Biological process	1.40E-10	83	648	13.86	5.71
GO:0045036	protein targeting to chloroplast	Biological process	1.50E-10	20	53	3.34	0.47
GO:0034641	cellular nitrogen compound metabolic process	Biological process	2.90E-10	104	905	17.36	7.97
GO:0044255	cellular lipid metabolic process	Biological process	6.10E-10	101	881	16.86	7.76

GO:0009523	photosystem II	Cellular component	7.20E-10	15	33	2.50	0.29
GO:0042440	pigment metabolic process	Biological process	1.80E-09	45	268	7.51	2.36
GO:0010027	thylakoid membrane organization	Biological process	2.10E-09	35	176	5.84	1.55
GO:0009668	plastid membrane organization	Biological process	2.10E-09	35	176	5.84	1.55
GO:0019748	secondary metabolic process	Biological process	2.70E-09	84	701	14.02	6.18
GO:0010207	photosystem II assembly	Biological process	3.10E-09	31	144	5.18	1.27
GO:0018130	heterocycle biosynthetic process	Biological process	3.60E-09	37	198	6.18	1.74
GO:0042793	transcription from plastid promoter	Biological process	3.90E-09	21	69	3.51	0.61
GO:0006066	alcohol metabolic process	Biological process	4.30E-09	91	793	15.19	6.99
GO:0006629	lipid metabolic process	Biological process	6.70E-09	110	1036	18.36	9.13
GO:0044271	cellular nitrogen compound biosynthetic process	Biological process	7.80E-09	69	542	11.52	4.78
GO:0031977	thylakoid lumen	Cellular component	1.20E-08	20	71	3.34	0.63
GO:0006733	oxidoreduction coenzyme metabolic process	Biological process	1.30E-08	38	217	6.34	1.91
GO:0035304	regulation of protein amino acid dephosphorylation	Biological process	1.50E-08	24	96	4.01	0.85
GO:0042646	plastid nucleoid	Cellular component	1.50E-08	12	24	2.00	0.21
GO:0035303	regulation of dephosphorylation	Biological process	2.40E-08	24	98	4.01	0.86
GO:0006790	sulfur metabolic process	Biological process	3.20E-08	57	424	9.52	3.74
GO:0019344	cysteine biosynthetic process	Biological process	4.40E-08	28	134	4.67	1.18
GO:0044283	small molecule biosynthetic process	Biological process	4.70E-08	110	1076	18.36	9.48
GO:0044446	intracellular organelle part	Cellular component	5.10E-08	196	2280	32.72	20.09
GO:0044422	organelle part	Cellular component	5.20E-08	196	2282	32.72	20.11
GO:0006534	cysteine metabolic process	Biological process	7.20E-08	28	137	4.67	1.21
GO:0055114	oxidation reduction	Biological process	8.50E-08	68	564	11.35	4.97
GO:0006740	NADPH regeneration	Biological process	8.50E-08	31	165	5.18	1.45
GO:0009773	photosynthetic electron transport in photosystem I	Biological process	9.40E-08	16	47	2.67	0.41
GO:0009070	serine family amino acid biosynthetic process	Biological process	1.10E-07	28	140	4.67	1.23
GO:0006739	NADP metabolic process	Biological process	1.30E-07	31	168	5.18	1.48
GO:0031399	regulation of protein modification process	Biological process	1.40E-07	24	107	4.01	0.94
GO:0044272	sulfur compound biosynthetic process	Biological process	1.90E-07	46	324	7.68	2.85
GO:0019438	aromatic compound biosynthetic process	Biological process	1.90E-07	50	368	8.35	3.24
GO:0022900	electron transport chain	Biological process	2.50E-07	22	94	3.67	0.83
GO:0006098	pentose-phosphate shunt	Biological process	2.60E-07	30	164	5.01	1.44
GO:0046496	nicotinamide nucleotide metabolic process	Biological process	2.70E-07	31	174	5.18	1.53
GO:0006769	nicotinamide metabolic process	Biological process	2.70E-07	31	174	5.18	1.53
GO:0009069	serine family amino acid metabolic process	Biological process	3.10E-07	31	175	5.18	1.54
GO:0019362	pyridine nucleotide metabolic process	Biological process	3.50E-07	31	176	5.18	1.55
GO:0005840	ribosome	Cellular component	4.30E-07	36	238	6.01	2.10
GO:0009820	alkaloid metabolic process	Biological process	6.00E-07	31	180	5.18	1.59
GO:0009072	aromatic amino acid family metabolic process	Biological process	6.40E-07	28	152	4.67	1.34
GO:0031978	plastid thylakoid lumen	Cellular component	6.50E-07	16	58	2.67	0.51
GO:0009543	chloroplast thylakoid lumen	Cellular component	6.50E-07	16	58	2.67	0.51
GO:0046906	tetrapyrrole binding	Molecular function	6.70E-07	28	137	4.67	1.21
GO:0043603	cellular amide metabolic process	Biological process	7.60E-07	31	182	5.18	1.60
GO:0009767	photosynthetic electron transport chain	Biological process	1.10E-06	18	70	3.01	0.62
GO:0006732	coenzyme metabolic process	Biological process	1.40E-06	48	370	8.01	3.26
GO:0009073	aromatic amino acid family biosynthetic process	Biological process	1.40E-06	18	71	3.01	0.63
GO:0046417	chorismate metabolic process	Biological process	1.80E-06	18	72	3.01	0.63
GO:0030095	chloroplast photosystem II	Cellular component	2.20E-06	8	14	1.34	0.12
GO:0009765	photosynthesis, light harvesting	Biological process	3.10E-06	11	27	1.84	0.24
GO:0009522	photosystem I	Cellular component	4.10E-06	8	15	1.34	0.13
GO:0000229	cytoplasmic chromosome	Cellular component	4.10E-06	8	15	1.34	0.13
GO:0009508	plastid chromosome	Cellular component	4.10E-06	8	15	1.34	0.13
GO:0006519	cellular amino acid and derivative metabolic process	Biological process	5.60E-06	78	762	13.02	6.71
GO:0000096	sulfur amino acid metabolic process	Biological process	5.60E-06	40	296	6.68	2.61
GO:0006725	cellular aromatic compound metabolic process	Biological process	7.00E-06	60	537	10.02	4.73
GO:0006520	cellular amino acid metabolic process	Biological process	7.60E-06	61	551	10.18	4.85
GO:0006633	fatty acid biosynthetic process	Biological process	1.10E-05	28	174	4.67	1.53
GO:0044106	cellular amine metabolic process	Biological process	1.10E-05	63	582	10.52	5.13
GO:0043648	dicarboxylic acid metabolic process	Biological process	1.10E-05	20	98	3.34	0.86
GO:0045893	positive regulation of transcription, DNA-dependent	Biological process	1.20E-05	40	306	6.68	2.70
GO:0045941	positive regulation of transcription	Biological process	1.20E-05	40	306	6.68	2.70
GO:0009055	electron carrier activity	Molecular function	1.40E-05	31	191	5.18	1.68
GO:0051254	positive regulation of RNA metabolic process	Biological process	1.50E-05	40	308	6.68	2.71
GO:0010628	positive regulation of gene expression	Biological process	1.60E-05	40	309	6.68	2.72
GO:0045935	positive regulation of nucleobase, nucleoside, nucleotide and nuclei	Biological process	2.00E-05	40	312	6.68	2.75
GO:0008652	cellular amino acid biosynthetic process	Biological process	2.10E-05	43	348	7.18	3.07
GO:0051173	positive regulation of nitrogen compound metabolic process	Biological process	2.10E-05	40	313	6.68	2.76

GO:0032268	regulation of cellular protein metabolic process	Biological process	2.10E-05	25	150	4.17	1.32
GO:0030529	ribonucleoprotein complex	Cellular component	2.50E-05	37	295	6.18	2.60
GO:0044281	small molecule metabolic process	Biological process	2.60E-05	198	2509	33.06	22.11
GO:0016109	tetraterpenoid biosynthetic process	Biological process	2.60E-05	19	95	3.17	0.84
GO:0044262	cellular carbohydrate metabolic process	Biological process	2.60E-05	105	1166	17.53	10.27
GO:0016117	carotenoid biosynthetic process	Biological process	2.60E-05	19	95	3.17	0.84
GO:0009891	positive regulation of biosynthetic process	Biological process	2.70E-05	45	376	7.51	3.31
GO:0031328	positive regulation of cellular biosynthetic process	Biological process	2.70E-05	45	376	7.51	3.31
GO:0010467	gene expression	Biological process	3.50E-05	170	2109	28.38	18.58
GO:0010557	positive regulation of macromolecule biosynthetic process	Biological process	3.70E-05	40	321	6.68	2.83
GO:0009308	amine metabolic process	Biological process	3.80E-05	64	621	10.68	5.47
GO:0019220	regulation of phosphate metabolic process	Biological process	4.00E-05	24	146	4.01	1.29
GO:0051174	regulation of phosphorus metabolic process	Biological process	4.00E-05	24	146	4.01	1.29
GO:0006636	unsaturated fatty acid biosynthetic process	Biological process	4.10E-05	14	56	2.34	0.49
GO:0033559	unsaturated fatty acid metabolic process	Biological process	4.10E-05	14	56	2.34	0.49
GO:0010287	plastoglobule	Cellular component	4.20E-05	13	53	2.17	0.47
GO:0003735	structural constituent of ribosome	Molecular function	4.40E-05	31	204	5.18	1.80
GO:0016108	tetraterpenoid metabolic process	Biological process	4.50E-05	19	99	3.17	0.87
GO:0016116	carotenoid metabolic process	Biological process	4.50E-05	19	99	3.17	0.87
GO:0009309	amine biosynthetic process	Biological process	4.70E-05	43	361	7.18	3.18
GO:0016114	terpenoid biosynthetic process	Biological process	4.80E-05	25	158	4.17	1.39
GO:0006470	protein amino acid dephosphorylation	Biological process	4.80E-05	24	148	4.01	1.30
GO:0031325	positive regulation of cellular metabolic process	Biological process	4.80E-05	45	386	7.51	3.40
GO:0042742	defense response to bacterium	Biological process	5.50E-05	32	235	5.34	2.07
GO:0051246	regulation of protein metabolic process	Biological process	5.80E-05	25	160	4.17	1.41
GO:0019825	oxygen binding	Molecular function	5.90E-05	15	59	2.50	0.52
GO:0010103	stomatal complex morphogenesis	Biological process	6.00E-05	18	92	3.01	0.81
GO:0009893	positive regulation of metabolic process	Biological process	6.10E-05	45	390	7.51	3.44
GO:0010604	positive regulation of macromolecule metabolic process	Biological process	6.90E-05	40	331	6.68	2.92
GO:0005975	carbohydrate metabolic process	Biological process	8.00E-05	116	1360	19.37	11.98
GO:0061024	membrane organization	Biological process	8.00E-05	44	382	7.35	3.37
GO:0016044	cellular membrane organization	Biological process	8.00E-05	44	382	7.35	3.37
GO:0009696	salicylic acid metabolic process	Biological process	8.10E-05	20	113	3.34	1.00
GO:0051656	establishment of organelle localization	Biological process	1.10E-04	18	96	3.01	0.85
GO:0016311	dephosphorylation	Biological process	1.20E-04	25	167	4.17	1.47
GO:0006721	terpenoid metabolic process	Biological process	1.30E-04	25	168	4.17	1.48
GO:0043228	non-membrane-bounded organelle	Cellular component	1.50E-04	61	632	10.18	5.57
GO:0043232	intracellular non-membrane-bounded organelle	Cellular component	1.50E-04	61	632	10.18	5.57
GO:0000097	sulfur amino acid biosynthetic process	Biological process	1.60E-04	29	214	4.84	1.89
GO:0009862	systemic acquired resistance, salicylic acid mediated signaling pathv	Biological process	1.70E-04	21	129	3.51	1.14
GO:0016556	mRNA modification	Biological process	1.70E-04	16	81	2.67	0.71
GO:0010310	regulation of hydrogen peroxide metabolic process	Biological process	1.90E-04	18	100	3.01	0.88
GO:0006396	RNA processing	Biological process	2.00E-04	67	699	11.19	6.16
GO:0080010	regulation of oxygen and reactive oxygen species metabolic proces	Biological process	2.10E-04	18	101	3.01	0.89
GO:0009533	chloroplast stromal thylakoid	Cellular component	2.50E-04	5	8	0.83	0.07
GO:0016491	oxidoreductase activity	Molecular function	2.50E-04	70	714	11.69	6.29
GO:0016138	glycoside biosynthetic process	Biological process	2.80E-04	19	113	3.17	1.00
GO:0009867	jasmonic acid mediated signaling pathway	Biological process	3.00E-04	23	155	3.84	1.37
GO:0051667	establishment of plastid localization	Biological process	3.00E-04	17	94	2.84	0.83
GO:0071395	cellular response to jasmonic acid stimulus	Biological process	3.00E-04	23	155	3.84	1.37
GO:0051644	plastid localization	Biological process	3.00E-04	17	94	2.84	0.83
GO:0009902	chloroplast relocation	Biological process	3.00E-04	17	94	2.84	0.83
GO:0009697	salicylic acid biosynthetic process	Biological process	3.10E-04	18	104	3.01	0.92
GO:0020037	heme binding	Molecular function	3.40E-04	20	116	3.34	1.02
GO:0016705	oxidoreductase activity, acting on paired donors, with incorporatio	Molecular function	3.40E-04	22	135	3.67	1.19
GO:0009814	defense response, incompatible interaction	Biological process	3.50E-04	35	295	5.84	2.60
GO:0009751	response to salicylic acid stimulus	Biological process	3.50E-04	29	224	4.84	1.97
GO:0009617	response to bacterium	Biological process	3.80E-04	37	321	6.18	2.83
GO:0031163	metallo-sulfur cluster assembly	Biological process	4.10E-04	16	87	2.67	0.77
GO:0016226	iron-sulfur cluster assembly	Biological process	4.10E-04	16	87	2.67	0.77
GO:0006412	translation	Biological process	5.00E-04	40	363	6.68	3.20
GO:0002376	immune system process	Biological process	5.20E-04	55	560	9.18	4.93
GO:0006955	immune response	Biological process	5.20E-04	55	560	9.18	4.93
GO:0009695	jasmonic acid biosynthetic process	Biological process	5.70E-04	15	80	2.50	0.70
GO:0009627	systemic acquired resistance	Biological process	6.00E-04	30	243	5.01	2.14
GO:0009753	response to jasmonic acid stimulus	Biological process	7.50E-04	29	234	4.84	2.06
GO:0044249	cellular biosynthetic process	Biological process	7.90E-04	270	3809	45.08	33.56

GO:0016137	glycoside metabolic process	Biological process	8.30E-04	21	144	3.51	1.27
GO:0016070	RNA metabolic process	Biological process	8.30E-04	153	2003	25.54	17.65
GO:0031323	regulation of cellular metabolic process	Biological process	8.60E-04	115	1433	19.20	12.63
GO:0000165	MAPKKK cascade	Biological process	9.80E-04	18	114	3.01	1.00
GO:0031348	negative regulation of defense response	Biological process	9.80E-04	21	146	3.51	1.29
GO:0031408	oxylipin biosynthetic process	Biological process	9.80E-04	15	84	2.50	0.74
GO:0009611	response to wounding	Biological process	9.80E-04	25	191	4.17	1.68
GO:0019761	glucosinolate biosynthetic process	Biological process	9.90E-04	16	94	2.67	0.83
GO:0016144	S-glycoside biosynthetic process	Biological process	9.90E-04	16	94	2.67	0.83
GO:0019758	glycosinolate biosynthetic process	Biological process	9.90E-04	16	94	2.67	0.83
GO:0009058	biosynthetic process	Biological process	1.00E-03	276	3926	46.08	34.59
GO:0006952	defense response	Biological process	1.30E-03	71	806	11.85	7.10
GO:0044275	cellular carbohydrate catabolic process	Biological process	1.40E-03	41	395	6.84	3.48
GO:0019222	regulation of metabolic process	Biological process	1.60E-03	122	1564	20.37	13.78
GO:0031407	oxylipin metabolic process	Biological process	1.60E-03	16	98	2.67	0.86
GO:0031090	organelle membrane	Cellular component	1.70E-03	76	909	12.69	8.01
GO:0010016	shoot morphogenesis	Biological process	1.80E-03	37	348	6.18	3.07
GO:0009863	salicylic acid mediated signaling pathway	Biological process	1.90E-03	23	176	3.84	1.55
GO:0045087	innate immune response	Biological process	1.90E-03	50	522	8.35	4.60
GO:0071446	cellular response to salicylic acid stimulus	Biological process	1.90E-03	23	176	3.84	1.55
GO:0016143	S-glycoside metabolic process	Biological process	1.90E-03	17	110	2.84	0.97
GO:0019757	glycosinolate metabolic process	Biological process	1.90E-03	17	110	2.84	0.97
GO:0019760	glucosinolate metabolic process	Biological process	1.90E-03	17	110	2.84	0.97
GO:0051640	organelle localization	Biological process	2.00E-03	18	121	3.01	1.07
GO:0005198	structural molecule activity	Molecular function	2.10E-03	32	269	5.34	2.37
GO:0007243	protein kinase cascade	Biological process	2.20E-03	18	122	3.01	1.07
GO:0005506	iron ion binding	Molecular function	2.20E-03	23	168	3.84	1.48
GO:0016168	chlorophyll binding	Molecular function	2.20E-03	7	19	1.17	0.17
GO:0016765	transferase activity, transferring alkyl or aryl (other than methyl) gr	Molecular function	2.20E-03	12	56	2.00	0.49
GO:0045730	respiratory burst	Biological process	2.30E-03	12	62	2.00	0.55
GO:0002679	respiratory burst during defense response	Biological process	2.30E-03	12	62	2.00	0.55
GO:0044085	cellular component biogenesis	Biological process	2.50E-03	75	884	12.52	7.79
GO:0009694	jasmonic acid metabolic process	Biological process	2.50E-03	15	92	2.50	0.81
GO:0046164	alcohol catabolic process	Biological process	2.50E-03	38	368	6.34	3.24
GO:0006399	tRNA metabolic process	Biological process	2.50E-03	17	113	2.84	1.00
GO:0006354	RNA elongation	Biological process	2.80E-03	13	73	2.17	0.64
GO:0009547	plastid ribosome	Cellular component	2.80E-03	5	12	0.83	0.11
GO:0009654	oxygen evolving complex	Cellular component	2.80E-03	6	18	1.00	0.16
GO:0010374	stomatal complex development	Biological process	3.10E-03	18	126	3.01	1.11
GO:0016052	carbohydrate catabolic process	Biological process	3.10E-03	41	412	6.84	3.63
GO:0010200	response to chitin	Biological process	3.10E-03	25	207	4.17	1.82
GO:0044444	cytoplasmic part	Cellular component	3.50E-03	350	5282	58.43	46.54
GO:0046365	monosaccharide catabolic process	Biological process	3.60E-03	37	362	6.18	3.19
GO:0009719	response to endogenous stimulus	Biological process	3.70E-03	67	781	11.19	6.88
GO:0006766	vitamin metabolic process	Biological process	4.70E-03	17	119	2.84	1.05
GO:0004033	aldo-keto reductase activity	Molecular function	4.80E-03	5	10	0.83	0.09
GO:0030001	metal ion transport	Biological process	5.20E-03	31	290	5.18	2.56
GO:0006007	glucose catabolic process	Biological process	5.20E-03	36	356	6.01	3.14
GO:0046483	heterocycle metabolic process	Biological process	5.40E-03	58	663	9.68	5.84
GO:0060255	regulation of macromolecule metabolic process	Biological process	5.40E-03	105	1362	17.53	12.00
GO:0019318	hexose metabolic process	Biological process	5.40E-03	42	438	7.01	3.86
GO:0019320	hexose catabolic process	Biological process	5.70E-03	36	358	6.01	3.15
GO:0071495	cellular response to endogenous stimulus	Biological process	5.70E-03	40	412	6.68	3.63
GO:0006351	transcription, DNA-dependent	Biological process	7.40E-03	96	1238	16.03	10.91
GO:0006350	transcription	Biological process	7.60E-03	96	1239	16.03	10.92
GO:0032774	RNA biosynthetic process	Biological process	7.60E-03	96	1239	16.03	10.92
GO:0000313	organellar ribosome	Cellular component	8.80E-03	5	15	0.83	0.13
GO:0004364	glutathione transferase activity	Molecular function	8.80E-03	6	17	1.00	0.15
GO:0034050	host programmed cell death induced by symbiont	Biological process	9.00E-03	25	223	4.17	1.96
GO:0009626	plant-type hypersensitive response	Biological process	9.00E-03	25	223	4.17	1.96
GO:0048522	positive regulation of cellular process	Biological process	9.70E-03	46	508	7.68	4.48
GO:0050832	defense response to fungus	Biological process	9.80E-03	22	187	3.67	1.65
GO:0043623	cellular protein complex assembly	Biological process	9.80E-03	35	356	5.84	3.14
GO:0080090	regulation of primary metabolic process	Biological process	1.00E-02	102	1345	17.03	11.85
GO:0048585	negative regulation of response to stimulus	Biological process	1.00E-02	22	188	3.67	1.66
GO:0006807	nitrogen compound metabolic process	Biological process	1.10E-02	218	3187	36.39	28.08
GO:0006855	multidrug transport	Biological process	1.10E-02	7	28	1.17	0.25

GO:0008219	cell death	Biological process	1.20E-02	29	280	4.84	2.47
GO:0016265	death	Biological process	1.20E-02	29	280	4.84	2.47
GO:0051707	response to other organism	Biological process	1.30E-02	64	779	10.68	6.86
GO:0042493	response to drug	Biological process	1.40E-02	7	29	1.17	0.26
GO:0034622	cellular macromolecular complex assembly	Biological process	1.40E-02	37	391	6.18	3.44
GO:0015893	drug transport	Biological process	1.40E-02	7	29	1.17	0.26
GO:0010218	response to far red light	Biological process	1.60E-02	11	67	1.84	0.59
GO:0006006	glucose metabolic process	Biological process	1.60E-02	38	409	6.34	3.60
GO:0043900	regulation of multi-organism process	Biological process	1.80E-02	11	68	1.84	0.60
GO:0048518	positive regulation of biological process	Biological process	1.80E-02	49	570	8.18	5.02
GO:0012501	programmed cell death	Biological process	1.80E-02	26	249	4.34	2.19
GO:0045088	regulation of innate immune response	Biological process	1.80E-02	24	222	4.01	1.96
GO:0051252	regulation of RNA metabolic process	Biological process	1.80E-02	85	1108	14.19	9.76
GO:0010114	response to red light	Biological process	1.80E-02	11	68	1.84	0.60
GO:0006355	regulation of transcription, DNA-dependent	Biological process	1.90E-02	84	1097	14.02	9.67
GO:0045426	quinone cofactor biosynthetic process	Biological process	2.00E-02	7	31	1.17	0.27
GO:0042375	quinone cofactor metabolic process	Biological process	2.00E-02	7	31	1.17	0.27
GO:0045449	regulation of transcription	Biological process	2.00E-02	84	1099	14.02	9.68
GO:0006631	fatty acid metabolic process	Biological process	2.00E-02	34	359	5.68	3.16
GO:0004185	serine-type carboxypeptidase activity	Molecular function	2.10E-02	6	20	1.00	0.18
GO:0016628	oxidoreductase activity, acting on the CH-CH group of donors, NA	Molecular function	2.10E-02	6	20	1.00	0.18
GO:0002682	regulation of immune system process	Biological process	2.20E-02	24	226	4.01	1.99
GO:0050776	regulation of immune response	Biological process	2.20E-02	24	226	4.01	1.99
GO:0009595	detection of biotic stimulus	Biological process	2.20E-02	10	60	1.67	0.53
GO:0042538	hyperosmotic salinity response	Biological process	2.20E-02	12	81	2.00	0.71
GO:0008152	metabolic process	Biological process	2.30E-02	420	6578	70.12	57.96
GO:0008187	poly-pyrimidine tract binding	Molecular function	2.30E-02	5	14	0.83	0.12
GO:0008266	poly(U) RNA binding	Molecular function	2.30E-02	5	14	0.83	0.12
GO:0009620	response to fungus	Biological process	2.40E-02	27	268	4.51	2.36
GO:0034621	cellular macromolecular complex subunit organization	Biological process	2.50E-02	37	407	6.18	3.59
GO:0009733	response to auxin stimulus	Biological process	2.60E-02	20	178	3.34	1.57
GO:0022621	shoot system development	Biological process	2.70E-02	39	437	6.51	3.85
GO:0051704	multi-organism process	Biological process	2.70E-02	72	928	12.02	8.18
GO:0048367	shoot development	Biological process	2.70E-02	39	437	6.51	3.85
GO:0009725	response to hormone stimulus	Biological process	2.80E-02	54	658	9.02	5.80
GO:0006655	phosphatidylglycerol biosynthetic process	Biological process	2.80E-02	9	52	1.50	0.46
GO:0071215	cellular response to abscisic acid stimulus	Biological process	3.00E-02	17	143	2.84	1.26
GO:0046471	phosphatidylglycerol metabolic process	Biological process	3.20E-02	9	53	1.50	0.47
GO:0010941	regulation of cell death	Biological process	3.30E-02	23	221	3.84	1.95
GO:0044282	small molecule catabolic process	Biological process	3.40E-02	52	635	8.68	5.59
GO:0009723	response to ethylene stimulus	Biological process	3.40E-02	19	170	3.17	1.50
GO:0031347	regulation of defense response	Biological process	3.50E-02	28	290	4.67	2.56
GO:0002252	immune effector process	Biological process	3.60E-02	19	171	3.17	1.51
GO:0009409	response to cold	Biological process	3.60E-02	33	360	5.51	3.17
GO:0006812	cation transport	Biological process	3.70E-02	38	432	6.34	3.81
GO:0009117	nucleotide metabolic process	Biological process	3.70E-02	43	505	7.18	4.45
GO:0000273	lipoic acid metabolic process	Biological process	3.70E-02	7	35	1.17	0.31
GO:0009106	lipoate metabolic process	Biological process	3.70E-02	7	35	1.17	0.31
GO:0051606	detection of stimulus	Biological process	3.80E-02	12	87	2.00	0.77
GO:0006753	nucleoside phosphate metabolic process	Biological process	3.80E-02	43	506	7.18	4.46
GO:0010363	regulation of plant-type hypersensitive response	Biological process	3.80E-02	21	198	3.51	1.74
GO:0010468	regulation of gene expression	Biological process	3.80E-02	93	1273	15.53	11.22
GO:0009738	abscisic acid mediated signaling pathway	Biological process	3.90E-02	16	135	2.67	1.19
GO:0004180	carboxypeptidase activity	Molecular function	3.90E-02	6	23	1.00	0.20
GO:0006612	protein targeting to membrane	Biological process	4.20E-02	22	213	3.67	1.88
GO:0043067	regulation of programmed cell death	Biological process	4.70E-02	22	215	3.67	1.89
GO:0070008	serine-type exopeptidase activity	Molecular function	4.70E-02	6	24	1.00	0.21
GO:0055086	nucleobase, nucleoside and nucleotide metabolic process	Biological process	4.80E-02	44	528	7.35	4.65

Supplementary Table S2. GO terms over-represented in the BTH-specific downregulated genes

GO accession	GO name	Ontology	FDR	Number of annoteted genes in the query set	Number of annoteted genes in the background set	Percentage of annoteted genes in the query set	Percentage of annotated genes in the background set
GO:0005576	extracellular region	Cellular component	1.10E-04	66	778	13.66	6.85
GO:0005886	plasma membrane	Cellular component	1.10E-04	130	1872	26.92	16.49
GO:0004674	protein serine/threonine kinase activity	Molecular function	1.30E-04	42	401	8.70	3.53
GO:0004672	protein kinase activity	Molecular function	1.50E-04	46	473	9.52	4.17
GO:0001883	purine nucleoside binding	Molecular function	5.50E-04	83	1117	17.18	9.84
GO:0001882	nucleoside binding	Molecular function	5.50E-04	83	1123	17.18	9.89
GO:0030554	adenyl nucleotide binding	Molecular function	5.50E-04	83	1116	17.18	9.83
GO:0030312	external encapsulating structure	Cellular component	5.60E-04	28	251	5.80	2.21
GO:0032559	adenyl ribonucleotide binding	Molecular function	7.10E-04	78	1051	16.15	9.26
GO:0005618	cell wall	Cellular component	1.00E-03	27	249	5.59	2.19
GO:0043565	sequence-specific DNA binding	Molecular function	1.00E-03	21	167	4.35	1.47
GO:0006468	protein amino acid phosphorylation	Biorogical process	1.30E-03	48	495	9.94	4.36
GO:0042398	cellular amino acid derivative biosynthetic process	Biorogical process	1.30E-03	31	264	6.42	2.33
GO:0030528	transcription regulator activity	Molecular function	1.90E-03	51	634	10.56	5.59
GO:0016773	phosphotransferase activity, alcohol group as acceptor	Molecular function	1.90E-03	47	572	9.73	5.04
GO:0003700	transcription factor activity	Molecular function	1.90E-03	49	606	10.14	5.34
GO:0043531	ADP binding	Molecular function	3.10E-03	8	33	1.66	0.29
GO:0016301	kinase activity	Molecular function	3.30E-03	49	624	10.14	5.50
GO:0017076	purine nucleotide binding	Molecular function	5.50E-03	84	1258	17.39	11.08
GO:0032553	ribonucleotide binding	Molecular function	8.30E-03	79	1190	16.36	10.48
GO:0032555	purine ribonucleotide binding	Molecular function	8.30E-03	79	1190	16.36	10.48
GO:0005524	ATP binding	Molecular function	8.30E-03	69	1011	14.29	8.91
GO:0006575	cellular amino acid derivative metabolic process	Biorogical process	1.50E-02	33	340	6.83	3.00
GO:0006952	defense response	Biorogical process	1.50E-02	62	806	12.84	7.10
GO:0006598	polyamine catabolic process	Biorogical process	1.50E-02	7	21	1.45	0.19
GO:0006270	DNA replication initiation	Biorogical process	1.70E-02	10	47	2.07	0.41
GO:0009698	phenylpropanoid metabolic process	Biorogical process	2.10E-02	22	193	4.55	1.70
GO:0016310	phosphorylation	Biorogical process	2.10E-02	49	610	10.14	5.37
GO:0006950	response to stress	Biorogical process	2.20E-02	137	2190	28.36	19.30
GO:0006595	polyamine metabolic process	Biorogical process	2.50E-02	8	33	1.66	0.29
GO:0015849	organic acid transport	Biorogical process	3.10E-02	18	149	3.73	1.31
GO:0004888	transmembrane receptor activity	Molecular function	3.40E-02	6	28	1.24	0.25
GO:0003677	DNA binding	Molecular function	3.90E-02	65	1005	13.46	8.85
GO:0016740	transferase activity	Molecular function	4.20E-02	92	1524	19.05	13.43
GO:0020037	heme binding	Molecular function	4.20E-02	13	116	2.69	1.02
GO:0016772	transferase activity, transferring phosphorus -containing groups	Molecular function	4.30E-02	52	776	10.77	6.84

Supplementary Table S3. Primers used in this study

Name	Sequence (5' to 3')	Target
**1) Fungal biomass quantification**		
Rs-1F	GCCTTTTCTACCTTAATTTGGCAG	*R. solani* AG-1, 1A rDNA
Rs-2R	GTGTGTAAATTAAGTAGACAGCAAATG	
BdFIM-F	CCTCACACGGATTTCGAGAGA	*Bradi2g13800, BdFIM*
BdFIM-R	GGACAACCCATTTCTGCGA	
**2) Gene expression analysis**		
W45L1-F	GGACACCTTCAGGGTGACAT	*Bradi2g30695, BdWRKY45L1*
W45L1-R	TTGTCGTCGTGGTAGGAGTG	
BdAOS-F	ACCGCCTGGACTTCTACTAC	*Bradi1g69330, BdAOS*
BdAOS-R	GAGGTTCTTCTTCTCCACCT	
rbcS1-F	TTTGCTTTGCGTTTTTCCTT	*Bradi4g08500, rbcS*
rbcS1-R	TGAACCAACACATACTCGACAG	
rbcS2-F	CGACTCTCCGATCAGACTCC	*Bradi4g08800, rbcS*
rbcS2-R	CATCACCGGTTCATTCCTCT	
Ubi4-F	TGACACCATCGACAACGTGA	*Bradi3g04730, Ubi4*
Ubi4-R	GAGGGTGGACTCCTTCTGGA	

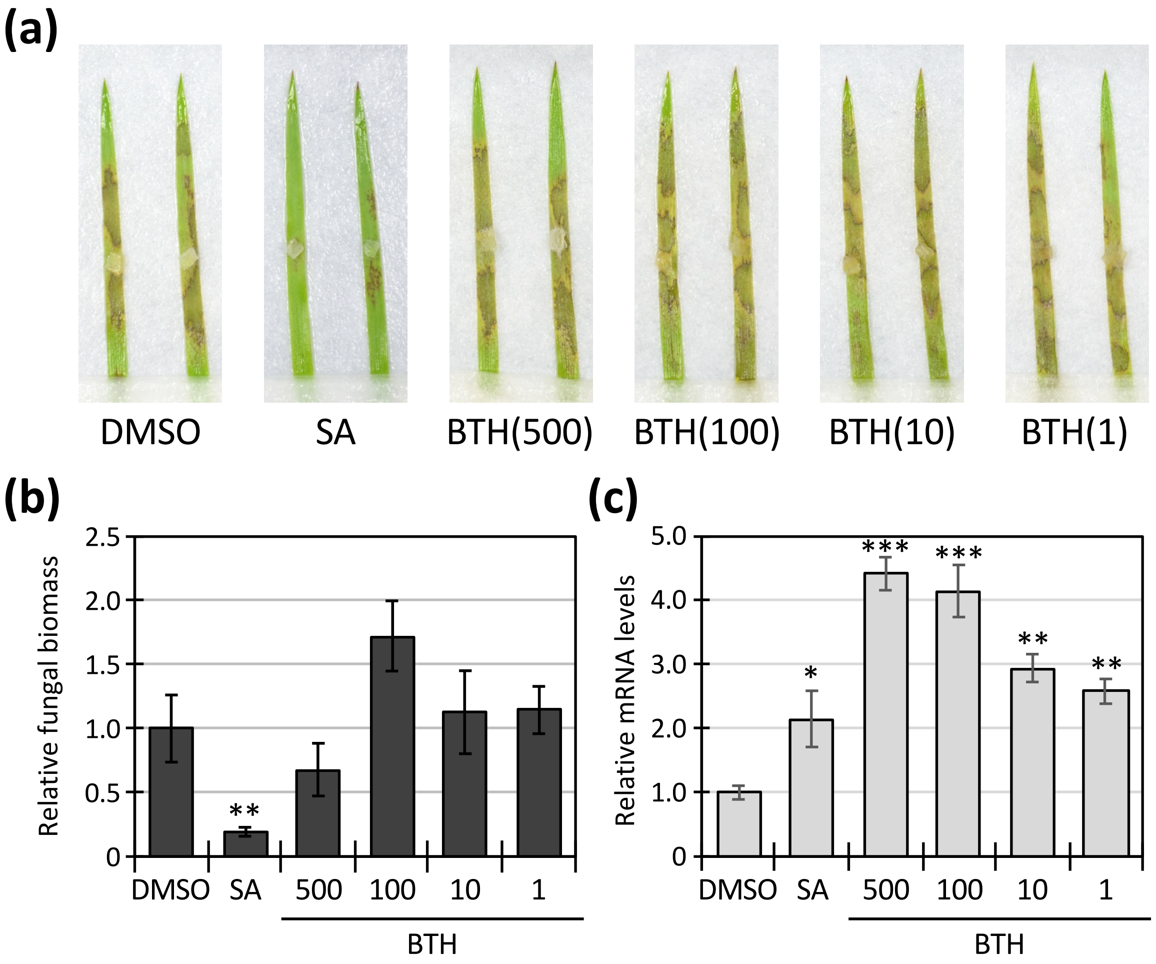

Supplementary Figure S1. BTH does not induce *Rhizoctonia solani* resistance in *Brachypodium distachyon* Bd21 at low doses

Detached leaves of *B. distachyon* Bd21 were spray-treated with 0.5% (v/v) DMSO, SA (500 μM), or BTH (500, 100, 10, or 1 μM) for 24 h. (a) Lesion formation and (b) relative biomass of *R. solani* in the leaves at 3 d post-inoculation. Data are presented as means ± SEM of values relative to the DMSO treatment, $n = 9$; **$P < 0.01$ using Student's *t*-tests. (c) Expression levels of a SA marker gene *BdWRKY45L1* (*Bradi2g30695*). Data are presented as means ± SEM of values relative to the DMSO treatment, $n = 6$; *$P < 0.05$, **$P < 0.01$, ***$P < 0.001$ using Student's *t*-tests. The experiments were performed twice with similar results and a representative result is shown.

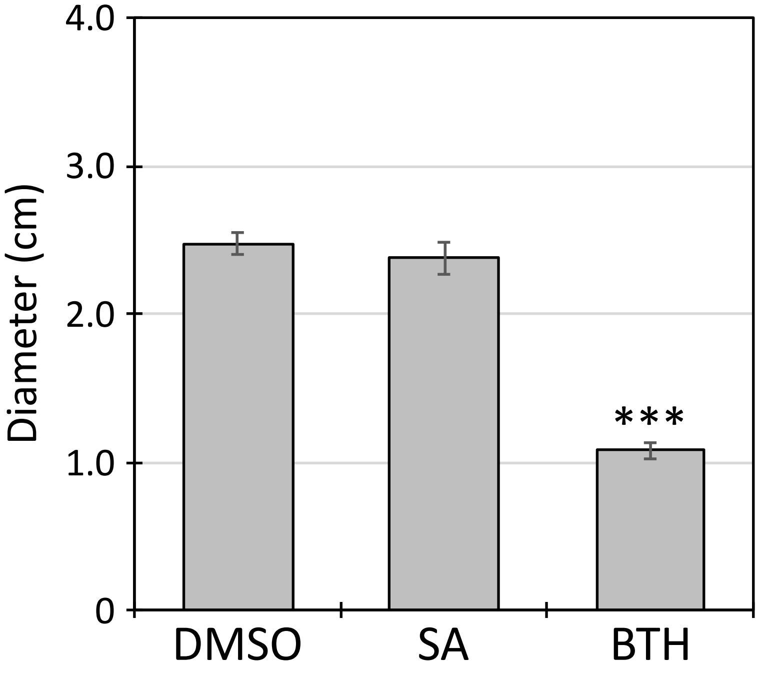

Supplementary Figure S2. Effects of BTH on the mycelial growth of *Rhizoctonia solani*

Mycelial plugs (3 mm diameter) bored from the edge of *R. solani* mycelia growing on PDA plates were inoculated onto the center of PDA plates containing 0.5% (v/v) DMSO, 0.1 mM SA, or 0.1 mM BTH. The growth of mycelia was measured at 24 h post-inoculation. Data are means ± SEM, n = 4; ***P < 0.001 using Student's t-tests. The experiments were performed twice with similar results and a representative result is shown.
